# Factors associated with different cannabis supply methods: results from the French 2017 ESCAPAD and Health Barometer surveys

**DOI:** 10.1186/s42238-025-00372-x

**Published:** 2026-01-08

**Authors:** Solène Wallez, Filiz Eren, Emmanuel Lahaie, Stanislas Spilka, Selma F. Rezag Bara, Gauthier Bayle, Bertrand Redonnet, Viet Nguyen-Thanh, Jean-Sébastien Cadwallader, Murielle Mary-Krause

**Affiliations:** 1 Sorbonne Université, INSERM, Institut Pierre Louis d’Epidémiologie et de Santé Publique, IPLESP, Equipe en Epidémiologie Sociale, Santé Mentale et Addictions, ESSMA, F75012, Paris, France; 2 The National Public Health Agency (Santé Publique France), Saint-Maurice, France; 3 French Monitoring Centre On Drugs and Drug Addiction (Observatoire Français Des Drogues Et Des Tendances Addictives - OFDT), Paris, 75007 France; 4 Department of General Practice, Sorbonne University, F75012, Paris, France

**Keywords:** Cannabis supply methods, Problematic cannabis use, Associated factors, Representative sample, France

## Abstract

**Background:**

Cannabis supply methods vary depending on the country, legislation, availability, and population characteristics. In many countries, cannabis consumption remains illegal and poses a major public health concern. Therefore, it is essential to study the factors associated with cannabis supply.

**Methods:**

Data were obtained from the 2017 French ESCAPAD and Health Barometer surveys (HBS). ESCAPAD is a national cross-sectional survey representative of 17-year-olds, while the HBS is a national cross-sectional telephone survey representative of French individuals aged 18–64. To ensure representativeness, data were weighted, and missing data were imputed. Three cannabis supply methods used in the past year were available in both databases and were analyzed using multivariate multinomial logistic regressions: (a) obtained for free; (b) bought from friends, relatives, or dealers; (c) home cultivation.

**Results:**

The study included 2,943 17-years-old and 1,221 adults who reported using at least one of the analyzed methods to obtain cannabis. The majority had purchased cannabis (60% in ESCAPAD and 68% in HBS), while 33% and 24%, respectively, obtained it free, and only 5% and 8% had cultivated it. Among both 17-year-olds and adults, compared with obtaining cannabis for free, being male and experiencing problematic cannabis use were associated with buying or cultivating cannabis. Among 17-year-olds, being an apprentice was associated with a higher likelihood of cultivation, while earning money in the past 30 days and experiencing depression were associated with buying cannabis. Early experimentation with cannabis was associated with both supply methods among adolescents. Among adults, the 26–34 age group was associated with both buying and cultivating, while having less than a high school diploma was associated only with purchasing.

**Conclusion:**

Cannabis supply methods are similar between minors and adults, with buying from friends, relatives, or dealers being the most common source. This study identifies vulnerable people who use cannabis and their acquisition practices, providing valuable insights for public policies.

**Supplementary Information:**

The online version contains supplementary material available at 10.1186/s42238-025-00372-x.

## Introduction

According to the 2022 United Nations World Drug Report (United Nations publication 2022), global drug use among individuals aged 15–64 increased by 26% in 2020 compared to the previous decade. Cannabis remains the most widely used drug worldwide, with 209 million users in the last twelve months of 2020, reflecting a 23% increase in cannabis use between 2010 and 2020 (United Nations publication 2022). In terms of disability-adjusted life years (DALYs), the disease burden due to cannabis use disorder (CUD) increased by 38.6% from 1990 to 2019, with males and young people aged 20–24 years experiencing the highest DALYs in 2019 (Shao et al. 2023).

A recent factor contributing to the rise in cannabis use may be the legalization of cannabis in some parts of the world (United Nations publication 2022), as it has become easier to access cannabis with the increasing availability of legal cannabis outlets since restrictions were lifted. Indeed, the legalization of cannabis appears to have led to an increase in daily consumption of this drug, with a marked rise in the reported frequent use of high-potency products among young adults (United Nations publication 2022). Fischer et al. described how, in Canada, where cannabis has been legalized since 2018 (Department of Justice 2018), adults who use cannabis transitioned from illegal to legal sources of cannabis products, while minors (e.g., those under the legal age of use) continued to have no difficulty accessing cannabis from illegal sources (Fischer et al. 2021). In Quebec, similar results were observed following legalization in 2018, with the proportion of consumers obtaining cannabis from illegal suppliers decreasing from 32% in 2018 to 11% in 2021, while the proportion obtaining it from the Société Québécoise Du Cannabis (SQDC, a government-owned monopoly on recreational cannabis sales) increased from 45 to 70% between 2019 and 2021 (Ministère de la Santé et des Services Sociaux du Québec 2023). In Los Angeles (USA), where cannabis was also legalized several years ago, the most common source of cannabis was recreational cannabis retailers (59.1%), while the least common source was a stranger or dealer (5.5%) (D’Amico et al. 2020).

However, many countries still maintain cannabis consumption as illegal, yet regular and problematic cannabis use remains an important public health concern. Although experimentation with the therapeutic use of cannabis in a controlled setting, limited to patients suffering from serious illnesses, began in France on March 26, 2021, and is set to continue until March 31, 2026 (Direction de l’information légale et administrative 2025), cannabis remains prohibited. It is classified as a narcotic, and all penalties applicable to narcotics apply. In particular, under Article 222–37 of the French Penal Code, the illegal transport, possession, supply, transfer, acquisition, or use of large quantities of narcotics is punishable by up to ten years' imprisonment and a fine of €7,500,000 (Légifrance 2025). Furthermore, under French law, the cultivation of cannabis is considered a serious trafficking offense, punishable more serverely than ‘traditional’ trafficking, by up to 20 years in prison and the same fine, which is also the penalty that applies to the illicit production or manufacture of narcotics (Légifrance 2025). Consuming cannabis in any form is also a criminal offense in France. Nevertheless, since 2020, if you are caught using drugs or in possession of small quantities, you may receive an on-the-spot fixed fine of €200, issued by the police or gendarmerie (Ministère de l’intérieur 2022). The offense is recorded on the criminal record. This procedure applies only to adults and only in cases of simple use (i.e., when no other offenses are involved). For minors, the law provides specific measures: authorities may summon the minor to appear before the juvenile court, where they may be sentenced to an educational measure aimed at fostering responsibility. Minors also face a prison sentence equal to half the term applicable to adults (Direction de l'information légale et administrative 2023).

In France, cannabis is primarily consumed as herb or resin (94%) in combination with tobacco (Santé publique France 2021; Le Nézet et al. 2021). Herb use has increased in recent years, unlike resin, as it is perceived as a more “natural” or “organic” product (Gandilhon et al. 2019), especially among young people, 67% of whom reported herb use during their last consumption in 2017 (Spilka et al. 2018). Resin, which is more accessible in terms of availability and price, tend to be used by younger and more socioeconomically vulnerable individuals, as well as heavier users (Gandilhon et al. 2019). Consumer preference vary according to their social environments, with a growing demand for high-quality, natural products, and safer supply sources. Concerns about contributing to criminal networks have encouraged the expansion of home cultivation and do-it-yourself production. This trend has been supported by the growth of online retailers providing access to cultivation equipment and information (Gandilhon et al. 2019). According to the 2010 Health Barometer, 6% of people who used cannabis, aged 15–64, reported consuming home-grown cannabis in the previous 12 months, compared with 46% who had purchased it and 71% who had received it free of charge (Beck et al. 2011). Men were more likely than women to purchase cannabis (52% vs. 36%) and to cultivate it themselves (7% vs. 3%), with no sex differences in receiving cannabis as a gift. Acquisition methods also varied by frequency of use: more than one in four daily users purchased cannabis exclusively, compared with fewer than one in ten occasional users (less than once a month). Self-cultivation accounted for 11% of daily users’ supply (1% exclusively). “Exclusive self-cultivation” was most common among less frequent users within the month (i.e., those reporting use fewer than 10 times per month). It is likely that, unlike regular or daily users, these individuals can more easily satisfy their consumption entirely through their own cultivation (Beck et al 2011).

Examining the cannabis purchasing patterns of minors and adults is crucial to understanding how cannabis consumption occurs and whether social inequalities exist. In the Netherlands, a survey conducted among people who use cannabis from seven European countries showed that the most common way to obtain cannabis was to buy it oneself. Unlike Dutch participants, who mainly bought cannabis in coffeeshops, the most common source of supply for French consumers was street dealers and delivery services (Skliamis & Korf 2022a). The authors note that only a small minority practiced home cultivation, which aligns with findings by Belackova et al., who reported that between 1 to 10% of people who use cannabis cultivate cannabis in countries where it is illegal (Belackova et al. 2019). In fact, reasons for cultivating cannabis at home include avoiding the illegal market, such as the dark web (Childs et al. 2022), obtaining better quality, and reducing costs (Belackova et al. 2019).

Self-cultivation has become increasingly popular since the 2000 s, with a boom in the 2010 s, driven by greater availability of seeds, online tutorials, and technologies such as LED lights. Furthermore, although purchasing cannabis online dates back to the early days of e-commerce, it gained significant momentum with the development of the Dark Web and cryptocurrencies, particularly with the emergence of Silk Road in 2011 (Martin 2014). Following the closure of several major Dark Web marketplaces, alternatives platforms have proliferated, leading to a rise in smaller-scale platforms. Since 2016, social networks and messaging apps (Telegram, Instagram, Snapchat) have become increasingly popular channels for selling cannabis, often facilitating local deliveries (Berg et al. 2023; Nali et al. 2024). Although online drug purchases increased during the COVID-19 pandemic, some international literature suggest that this largely reflected temporary market adjustment (Hawdon et al. 2022). In France, online purchasing of cannabis was marginal in 2017 (2.2%), and no national data are available for the period during or after the pandemic. The 2020 Cannabis Online Survey, conducted two months after the end of the first lockdown, documented changes in use and supply patterns but did not directly measure online purchasing (Brissot et al. 2020). Regardless of the mode of contact, transactions generally involve a dealer and therefore fall within the broader category of dealer-based supply.

Current findings in the literature indicate that supply practices among people who use cannabis vary considerably depending on factors such as country of residence (Azofeifa et al. 2021), cannabis legislation (Wadsworth et al. 2022a, b), and drug availability (Hakkarainen & Perälä, 2016; Wadsworth et al. 2021). Therefore, it is important to examine the determinants of cannabis procurement methods to determine whether certain supply sources are preferred within specific demographic subgroups. Studies have shown that the method of cannabis procurement varies according to sex (Azofeifa et al. 2021; Cristiano et al. 2022; D’Amico et al. 2020; King et al. 2016; Kolar 2021; Skliamis & Korf, 2022a), age (Cristiano et al. 2022; King et al. 2016; Skliamis & Korf, 2022a; United Nations publication 2022), marital status (Cristiano et al. 2022), household type (Skliamis & Korf, 2022a), education level (Cristiano et al. 2022), and cannabis consumption habits (Azofeifa et al. 2021; Cristiano et al. 2022; King et al. 2016; Wadsworth et al. 2022a, b). Furthermore, it is worthwhile to consider the association between socioeconomic status and neighborhood characteristics with cannabis supply methods, as cannabis sales tend to be concentrated in disadvantaged neighborhoods (Williams et al. 2023). However, existing studies have mostly been conducted in locations where cannabis is legalized (Azofeifa et al. 2021; D’Amico et al. 2020; Skliamis & Korf, 2022a; Williams et al. 2023), focusing on cannabis cultivation as a supply method or on specific age groups. Evidence regarding this association is limited in the current literature, particularly in countries where cannabis is not legalized, and comparisons between young people and adults regarding cannabis supply methods appear to be a novel area of research.

In this context, the main objective of our study is to investigate the different methods of cannabis supply in France by examining user buying practices and identifying the factors associated with various modes of supply. We aim to examine the role of individual socioeconomic position and residential context on cannabis supply, hypothesizing that dealing is the primary supply method among individuals of lower socioeconomic status and that cultivation predominates among those with higher levels of cannabis consumption. France presents an ideal setting for our research, given the rapid expansion of the illicit cannabis market, the ineffectiveness of law enforcement efforts to curb trafficking, and the relatively lenient penalties for consumers, which fail to deter further market growth.

## Methods

### Study design

To achieve these objectives, we used data from two complementary surveys: the 2017 ESCAPAD survey, targeting the general French population of 17-year-olds, and the 2017 French National Health Barometer, which included participants aged 18–64.

#### The 2017 ESCAPAD survey

**Fig. 1:**
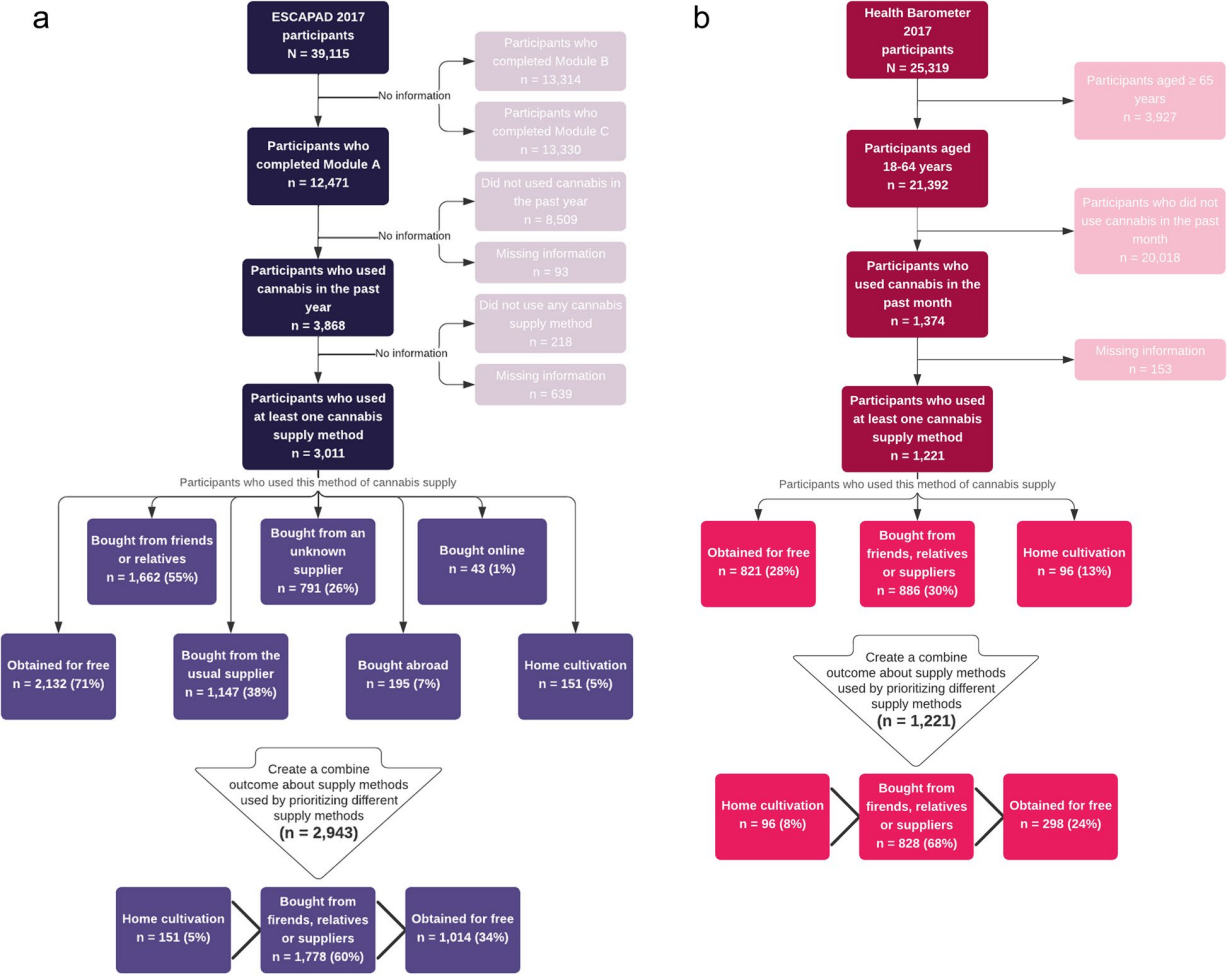
a. Study population flowchart from the 2017 ESCAPAD survey. **b**. Study population flowchart from the 2017 Health Barometer

Since 2000, the French Monitoring Centre on Drugs and Drug Addiction (Observatoire français des drogues et des tendances addictives, OFDT) has conducted regular surveys among all 17-year-olds in France during the mandatory National Defense and Citizenship Day (NDCD), known as ESCAPAD (Enquête sur la santé et les consommations lors de la journée d’appel et de préparation à la défense) (OFDT 2019). Attendance at this one-day session is mandatory for all French adolescents and is a prerequisite for participating in national education and university exams, as well as obtaining a driver's license. This requirement ensures very high participation and excellent representativeness of the entire population of 17-year-old French adolescents. The 8th wave of this survey took place in March 2017, with 39,115 youths completing a voluntary, paper-based, self-administered questionnaire on their health and use of psychoactive substances. Each questionnaire consisted of a short core section supplemented by three specific modules (labeled A, B, and C), each addressing a particular theme. Each respondent was given only one of the three versions of the questionnaires, selected at random with equal probability (the questionnaires were delivered to the centers in packs of 50 containing the three modules in rotation). Participants were gathered in large rooms with dozens or even hundreds of people, overseen by a single supervisor. After receiving information about and a presentation of the ESCAPAD study, participants had the option not to take part in the survey, as it was not mandatory. All those present received a questionnaire, which they could choose not to complete, but they were required to remain in the room while it was being administered. Completed questionnaires were placed in envelopes and sealed by the participants themselves. Blank questionnaires were collected at the end, along with the completed ones. The questionnaire took approximately 20 min to complete, and complete anonymity was guaranteed, resulting in a response rate of over 98%. Among the respondents, 12,471 individuals completed the module focusing on cannabis supply methods. Of these, 3,868 participants had used cannabis in the past year, and 2,943 reported using one of the combined and prioritized methods of cannabis acquisition analyzed in this study (Fig. 1a).

#### The 2017 Health Barometer

Data were obtained from the 2017 national French “Health Barometer”, a cross-sectional phone survey conducted by the French National Public Health Agency (Santé Publique France, SPF) to investigate the health-related behaviors and opinions of the French population aged 18–85 (Santé publique France 2023). The survey used telephone and computer-assisted collection (CATI) interviewing based on two-stage random sampling (household, individual), with mobile and landline phone numbers randomly generated (Richard et al. 2018). From January 5th to July 18th, 2017, participants were selected from households, and the first person to answer via landline or mobile phone was included. Up to 25 attempts were made to contact potential participants to maximize inclusion. In total, 25,319 individuals were included (9,717 via landline and 15,602 via mobile phone). Regarding illicit substance use, the questionnaire was administered to 21,392 individuals aged 18–64. Among them, 1,374 had used cannabis in the past month, and 1,221 reported using at least one cannabis supply method in the last 30 days and were included in this study (Fig. 1b).

### Measures

#### Outcome: cannabis supply methods

In the 2017 ESCAPAD survey, cannabis supply methods over the past 12 months were assessed using the question: “In the past 12 months, how did you obtain cannabis for your personal consumption?” Seven non-exclusive supply methods were proposed: obtained for free, bought from friends or relatives, bought from a regular supplier, bought from an unknown supplier, bought from abroad, bought online, and home cultivation (Fig. 1a). For each supply method, the reported frequency, ranging from 0 times to more than 10 times, was dichotomized into “Yes” or “No” for comparison with the 2017 Health Barometer.

In the 2017 Health Barometer, participants who reported cannabis use in the past 30 days were asked about their cannabis supply method in the past year using the following questions: “In the past 12 months, have you 1) bought cannabis, 2) obtained cannabis for free, 3) cultivated cannabis?”. Each question required a yes or no response (Fig. 1b).

From the three cannabis supply methods reported in both databases, the outcome was categorized as follows: “obtained for free”, “bought from friends, relatives, or suppliers”, and “home cultivation”. If a participant used multiple cannabis supply methods, considering that the method may evolve with consumption levels – initially receiving cannabis for free during the initiation of use, then transitioning to buying as consumption increases and it becomes less feasible to obtain it for free, and ultimately resorting to cultivation for those with higher consumption levels (Azofeifa et al. 2021; Cristiano et al. 2022; Wadsworth et al. 2022a, b) – we categorized based on the assumption that home cultivation of cannabis takes precedence over buying cannabis, which in turn takes precedence over obtaining cannabis for free.

Three sensitivity analysis were performed. The first, across both databases, focused on validating the classification of individuals: those who purchased cannabis only once or twice in the last month were categorized in the “obtained for free” group if they also acquired it for free. The other two sensitivity analyses were specific to the ESCAPAD database: the first distinguished between buying cannabis from friends or relatives and buying it from suppliers, and the second included only those individuals who consumed cannabis during the month to align with the data available in the Health Barometer survey.

#### Potential associated factors

##### Sociodemographic characteristics

Covariates included sex (“Male”, “Female”), age categorized into three groups (“18–25 years old”, “26–34 years old”, “ ≥ 35 years old”) (only in the Health Barometer, as all participants in ESCAPAD were 17 years old), educational status in ESCAPAD (“Middle school, high school or higher education”, “Apprenticeship”, “No longer in education system”) or highest diploma in the Health Barometer (“ ≤ High school diploma”, “ > High school diploma”), and whether a participant had repeated a school year (“No”, “Yes”). We also used the 2015 French DEPrivation index (FDep), which we created at the departmental level. This index provides a geographic indicator of social disadvantage specifically adapted for health studies in the French population, categorized into quintiles as described elsewhere (Rey et al. 2009; Windenberger et al. 2012). Agglomeration size was also considered and categorized into four groups: “Less than 2,000 inhabitants”, “From 2,000 to 19,999 inhabitants”, “From 20,000 to 199,999 inhabitants”, and “200,000 inhabitants and over” (Insee 2021).

In ESCAPAD, participants were asked about their living situation with parents or only one parent (“Yes”, “No”). The parental socio-professional category was reported for both parents and determined by the highest occupational grade of either parent, categorized as “Artisans, executives, farmers, traders”, “Intermediate professions or employees”, “Manual workers”, or “No profession”. Additionally, participants were asked whether they had received any money in the past 30 days, including pocket money, salary, or apprenticeship pay (“No”, “Yes”).

In the Health Barometer, participants were asked about their living situation with children under 15 years old (“No”, “Yes”). Their socio-professional category was recoded as “Artisans, executives, farmers, traders”, “Intermediate professions or employees”, or “Manual workers”, and their monthly income was recoded as “ ≥ 1500 € per month” or “ < 1500 € per month”.

##### Physical and mental Health

Health status was assessed across four categories, which were regrouped into three categories due to small sample sizes by combining the two lower conditions (“Very satisfactory”, “Somewhat satisfactory”, “Not at all or not very satisfactory”).

In ESCAPAD, mental health was evaluated using the Adolescent Depression Rating Scale (ADRS), a validated measure for assessing depression in adolescents aged 13 to 20 years. A 10-item self-report version was used, and dichotomized with a cut-off of four, corresponding to the DSM-IV criteria (“No depression”, “Depression”) (Revah-Levy et al. 2007).

In the Health Barometer, mental health was evaluated using the short version of the *Composite International Diagnostic Interview* (CIDI-SF) (Gigantesco & Morosini 2008), which measures Characterized Depressive Episodes (CDE) (Sapinho et al. 2008). A CDE is defined by a period of 15 days of sadness or loss of interest every day; along with the presence of at least three secondary symptoms (“No depression”, “Depression”).

##### Cannabis use

Age at first initiation of cannabis (“Late initiation (> 16 years old)”, “Early initiation (≤ 16 years old)”), cannabis use in the past month (“No”, “Not regular (Less than 10 times)”, “Regular (10 times or more)”, “Daily”), and the Cannabis Abuse Screening Test (CAST) (Legleye et al., 2015), which consists of six Likert-type items assessing the frequency of events within the past 12 months, were used to identify the risk of problematic cannabis use (“No”, “Yes”), with a cut-off of seven.

### Statistical analysis

To ensure the representativeness of the 2017 French population, the data were weighted using information from the National Institute of Statistics and Economic Studies (Insee 2017). The ESCAPAD sample was adjusted to match regional weights and intra-regional sex ratios, and the cannabis supply module was also weighted according to three indicators: daily tobacco use, regular alcohol use, and regular cannabis use. The Health Barometer was adjusted to align with sex stratified by age in ten-year groups, urban unit size, region of residence, level of education, and household size.

For both databases, participants’ characteristics were described based on the method of cannabis supply: “Obtained for free”, “Bought from friends, relatives, or suppliers”, or “Home cultivation”. Statistical differences between the three groups were assessed using either the chi-squared test with Rao & Scott's second-order correction or the Wilcoxon rank-sum test for complex survey samples.

Multinomial logistic regression modelling was employed to identify factors associated with the different cannabis supply methods. Factors hypothesized to be associated with cannabis supply methods were tested in univariate models. Variables with a *p*-value < 0.2 were included in the multivariate model.

In ESCAPAD, the average percentage of missing data for the selected covariates was 1.8% (ranging from 0.1% for sex to 5.4% for parental socio-professional category). In the 2017 Health Barometer, the average percentage of missing data for selected covariates were 0.5% (ranging from 0.08% for highest diploma to 3.4% for monthly income). To ensure the inclusion of all participants who had used the studied modes of supply, missing values for all covariates were imputed using *missForest*, which has demonstrated effectiveness in handling missing data, particularly in datasets with various types of variables, and the lowest imputation error for categorical variables (Waljee et al. 2013; Stekhoven & Bühlmann 2012).

Correlation and interactions between selected variables were tested.

Data processing and analyses were performed using SAS® 9.4 and R 4.1.0 software (R Core Team 2020) (packages *survey* (Thomas Lumley 2020) and *missForest* (Stekhoven 2022)).

## Results

### Participant’s description

Tables 1 and 2 describe the characteristics of the participants. The ESCAPAD participants had a median age of 17.3 years, with 55% being male. Most were enrolled in middle school, high school, or higher education (88.8%), and the majority lived with their parents (89.4%), with 53.8% of parents holding executive-level occupations. In the Health Barometer survey, 71.9% of participants were male, with a median age of 28 years. The majority had an educational level equivalent to or lower than a high school degree (71.6%), resided in urban areas with over 200,000 inhabitants (56.1%), and 30.3% were manual workers.

**Table 1:** Characteristics of ESCAPAD 2017 participants included in the study (module A, people who have used cannabis in the past year, *n* = 2,943) by the three methods of cannabis supply

Variable, *n (%)*	Overall (*n* = 2,943)	Obtained for free (*n* = 1,014)	Bought from friends, relatives or suppliers (*n* = 1,778)	Home cultivation (*n* = 151) *p*-value^1^	
Sociodemographic characteristics					
Sex					< 0.001
Male	1,617 (55.0)	467 (46.1)	1,031 (58.1)	119 (79.5)	
Female	1,321 (45.0)	546 (53.9)	745 (41.9)	31 (20.5)	
Unknown	4	1	2	1	
Age, median (IQR)	17.35 (17.27–17.69)	17.35 (17.18–17.60)	17.35 (17.18–17.69)	17.43 (17.27–17.85)	< 0.001
Unknown	39	14	22	4	
Educational status					< 0.001
Middle school, high school or higher education	2,599 (88.8)	947 (93.6)	1,547 (87.6)	105 (70.1)	
Apprenticeship	217 (7.4)	44 (4.3)	147 (8.3)	26 (17.6)	
No longer in education system	112 (3.8)	22 (2.1)	72 (4.1)	19 (12.3)	
Unknown	14	2	12	1	
Repeating a school year	843 (28.7)	232 (22.8)	545 (30.7)	67 (44.4)	< 0.001
Unknown	5	0	5	0	
2015 French DEPrivation index (FDep)					0.030
Q1 (most favored)	568 (19.5)	227 (22.7)	323 (18.4)	18 (12.0)	
Q2	708 (24.4)	241 (24.2)	426 (24.2)	41 (27.3)	
Q3	561 (19.3)	193 (19.3)	342 (19.4)	26 (17.2)	
Q4	553 (19.0)	181 (18.1)	335 (19.0)	37 (24.4)	
Q5 (most disadvantaged)	518 (17.8)	156 (15.7)	333 (18.9)	29 (19.1)	
Unknown	34	15	19	0	
Agglomeration size					0.134
Less than 2,000 inhabitants	595 (20.8)	210 (21.1)	348 (20.3)	37 (25.9)	
From 2,000 to 19,999 inhabitants	539 (18.9)	166 (16.6)	344 (20.1)	29 (20.7)	
From 20,000 to 199,999 inhabitants	554 (19.4)	188 (18.8)	340 (19.8)	27 (18.8)	
200,000 inhabitants and over	1,167 (40.9)	434 (43.5)	684 (39.9)	49 (34.6)	
Unknown	87	16	61	9	
Living with parents or one parent	2,612 (89.4)	915 (90.8)	1,570 (88.8)	127 (86.6)	0.159
Unknown	20	6	10	4	
Parental socio-professional category					0.273
Artisans, executives, farmers, traders	1,498 (53.8)	542 (55.8)	884 (52.9)	71 (51.1)	
Intermediate professions or employees	991 (35.6)	343 (35.3)	598 (35.8)	50 (35.6)	
Manual workers	220 (7.9)	68 (7.0)	140 (8.4)	12 (8.3)	
No profession	73 (2.6)	19 (1.9)	47 (2.8)	7 (5.0)	
Unknown	160	42	107	11	
Money in the past 30 days	2,592 (90.0)	874 (87.4)	1,595 (91.9)	122 (86.1)	0.001
Unknown	64	14	41	8	
Physical and mental health					
Health status					< 0.001
Somewhat satisfactory	1,502 (51.3)	454 (44.7)	962 (54.5)	86 (58.3)	
Very satisfactory	1,182 (40.4)	500 (49.4)	643 (36.4)	39 (26.1)	
Not at all or not very satisfactory	244 (8.3)	60 (5.9)	161 (9.1)	23 (15.5)	
Unknown	14	0	12	2	
Adolescent Depression Rating Scale (ADRS)					0.002
No depression	2,017 (72.2)	745 (76.4)	1,175 (70.0)	96 (69.0)	
Depression	777 (27.8)	230 (23.6)	503 (30.0)	43 (31.0)	
Unknown	149	38	100	11	
Cannabis use					
Age of cannabis experimentation					< 0.001
Late initiation (> 16 years old)	352 (12.2)	215 (21.6)	134 (7.7)	2 (1.4)	
Early initiation (≤ 16 years old)	2,534 (87.8)	783 (78.4)	1,612 (92.3)	139 (98.6)	
Unknown	56	15	32	9	
Cannabis use in the past month					< 0.001
No	825 (28.0)	489 (48.3)	321 (18.1)	15 (9.9)	
Not regular (Less than 10 times)	1,394 (47.4)	500 (49.3)	858 (48.3)	36 (23.7)	
Regular (10 times or more)	382 (13.0)	20 (1.9)	329 (18.5)	33 (21.8)	
Daily	341 (11.6)	5 (0.5)	269 (15.2)	67 (44.6)	
Problematic use of cannabis (advanced CAST)	712 (25.4)	44 (4.6)	569 (33.4)	99 (67.8)	< 0.001
Unknown	143	64	74	5	
Duration of cannabis use in years, median (IQR)	2.10 (1.27–2.85)	1.35 (1.10–2.27)	2.27 (1.35–3.10)	3.17 (2.18–4.40)	< 0.001
Unknown	100	30	57	13	

^1^Wilcoxon rank-sum test for complex survey samples; chi-squared test with Rao & Scott's second-order correction

**Table 2:** Characteristics of the Health Barometer 2017 participants included in the study (people who have used cannabis in the past 30 days, *n* = 1,221) by the three methods of cannabis supply

Variable, *n (%)*	Overall (*n* = 1,221)	Obtained for free (*n* = 298)	Bought from friends, family or suppliers (*n* = 828)	Home cultivation (*n* = 96)	*p*-value^1^
Sociodemographic characteristics					
Sex					0.002
Male	878 (71.9)	182 (61.2)	618 (74.7)	78 (80.9)	
Female	343 (28.1)	115 (38.8)	210 (25.3)	18 (19.1)	
Unknown	0	0	0	0	
Age					0.010
18–25 years old	471 (38.6)	123 (41.3)	327 (39.5)	21 (22.4)	
26–34 years old	363 (29.7)	67 (22.6)	265 (32.0)	31 (32.1)	
≥ 35 years old	387 (31.7)	107 (36.1)	236 (28.5)	44 (45.5)	
Unknown	0	0	0	0	
Highest diploma					< 0.001
≤ High school diploma	874 (71.6)	182 (61.2)	616 (74.5)	76 (78.9)	
> High school diploma	346 (28.4)	115 (38.8)	211 (25.5)	20 (21.1)	
Unknown	1	0	1	0	
Repeating a school year	393 (58.4)	81 (51.7%)	290 (59.5)	23 (76.3)	0.120
Unknown	548	142	340	66	
2015 French DEPrivation index (FDep)					0.112
Q1 (most favored)	262 (21.5)	74 (24.8)	180 (21.7)	9 (9.1)	
Q2	333 (27.3)	74 (24.9)	225 (27.2)	34 (35.7)	
Q3	225 (18.4)	57 (19.2)	153 (18.5)	15 (15.5)	
Q4	191 (15.6)	56 (18.9)	116 (14.0)	19 (19.4)	
Q5 (most disadvantaged)	210 (17.2)	36 (12.2)	154 (18.6)	19 (20.3)	
Unknown	0	0	0	0	
Agglomeration size					< 0.001
Less than 2,000 inhabitants	185 (15.2)	54 (18.1)	97 (11.8)	35 (36.2)	
From 2,000 to 19,999 inhabitants	173 (14.3)	42 (14.4)	117 (14.2)	14 (14.2)	
From 20,000 to 199,999 inhabitants	175 (14.4)	38 (12.9)	128 (15.6)	8 (8.6)	
200,000 inhabitants and over	682 (56.1)	161 (54.6)	481 (58.4)	39 (41.0)	
Unknown	6	2	4	0	
Living with children under 15 years old	370 (30.3)	88 (29.6)	233 (28.2)	49 (51.3)	0.003
Unknown	0	0	0	0	
Socio-professional category					0.188
Artisans, executives, farmers, traders	240 (20.3)	65 (22.8)	152 (19.0)	23 (24.3)	
Intermediate professions or employees	582 (49.3)	144 (50.2)	406 (50.7)	33 (35.2)	
Manual workers	358 (30.3)	77 (27.0)	243 (30.3)	38 (40.5)	
Unknown	41	11	27	2	
Monthly income					0.922
≥ 1500 € per month	806 (68.3)	194 (68.1)	550 (68.7)	62 (65.9)	
< 1500 € per month	374 (31.7)	91 (31.9)	251 (31.3)	32 (34.1)	
Unknown	41	13	26	2	
Physical and mental Health					
Health status					0.183
Not at all or not very satisfactory	56 (4.6)	19 (6.5)	31 (3.7)	6 (6.1)	
Somewhat satisfactory	735 (60.2)	165 (55.3)	503 (60.8)	68 (71.0)	
Very satisfactory	430 (35.2)	114 (38.2)	294 (35.5)	22 (23.0)	
Unknown	0	0	0	0	
Characterized depressive episode (CDE)					0.231
No depression	994 (81.6)	255 (85.7)	665 (80.7)	74 (77.0)	
Depression	224 (18.4)	43 (14.3)	160 (19.3)	22 (23.0)	
Unknown	3	0	3	0	
Cannabis use					
Age of cannabis experimentation					
Late initiation (> 16 years old)	573 (46.9)	166 (55.8)	372 (44.9)	35 (36.4)	0.013
Early initiation (≤ 16 years old)	648 (53.1)	132 (44.2)	456 (55.1)	61 (63.6)	
Unknown	0	0	0	0	
Cannabis use in the past month					< 0.001
Not regular (Less than 10 times)	483 (40.0)	231 (77.7)	236 (29.0)	16 (16.7)	
Regular (10 times or more)	285 (23.6)	42 (14.1)	222 (27.3)	21 (21.9)	
Daily	439 (36.4)	24 (8.2)	356 (43.8)	59 (61.4)	
Unknown	15	1	14	0	
Problematic use of cannabis (advanced CAST)	480 (39.3)	36 (12.0)	395 (47.7)	50 (51.8)	< 0.001
Unknown	0	0	0	0	
Duration of cannabis use in years, median (IQR)	12.0 (5.0–20.0)	12.0 (5.0–21.0)	12.0 (5.0–19.0)	19.0 (11.0–23.0)	< 0.001
Unknown	0	0	0	0	

^1^Wilcoxon rank-sum test for complex survey samples; chi-squared test with Rao & Scott's second-order correction

### Cannabis use and supply method

The majority of participants in the ESCAPAD and Health Barometer surveys reported buying cannabis (60% and 68%, respectively), while almost a third obtained it for free (33% and 24%), and only a small percentage had grown it themselves (5% and 8%) (Figs. 1a and b). Adolescents who reported receiving cannabis for free were more often girls, whereas those who reported buying or cultivating it were more often boys (Table 1). Adolescents who reported relying on home-cultivation were more frequently less advantaged (43.7%) than more advantaged adolescents (39.1%), whereas the pattern was reversed among adults (44.8% among more advantaged individuals and 39.6% among less advantaged adults) (Tables 1 and 2). Among 17-year-olds who had used cannabis in the past month, 11.6% reported daily use. Among adults who had used cannabis in the past 30 days, 36.4% reported daily use (Tables 1 and 2). Among people who reported home cultivation, people with daily use were the most frequent, regardless of age (44.6% among 17-year-olds and 61.4% among adults), whereas among individuals who reported obtaining cannabis for free or buying it, most were occasional or non-regular users, regardless of age (97.5% and 66.3% among 17-year-olds, and 91.3% and 55.3% among adults, respectively) (Tables 1 and 2). Apprentices more frequently reported daily cannabis use than participants in middle school, high school, or higher education (25.0% vs. 9.4%, *p* < 0.001), as well as problematic cannabis use (43.7% vs. 22.2%, *p* < 0.001).

### Factors associated with different cannabis supply methods

**Fig. 2:**
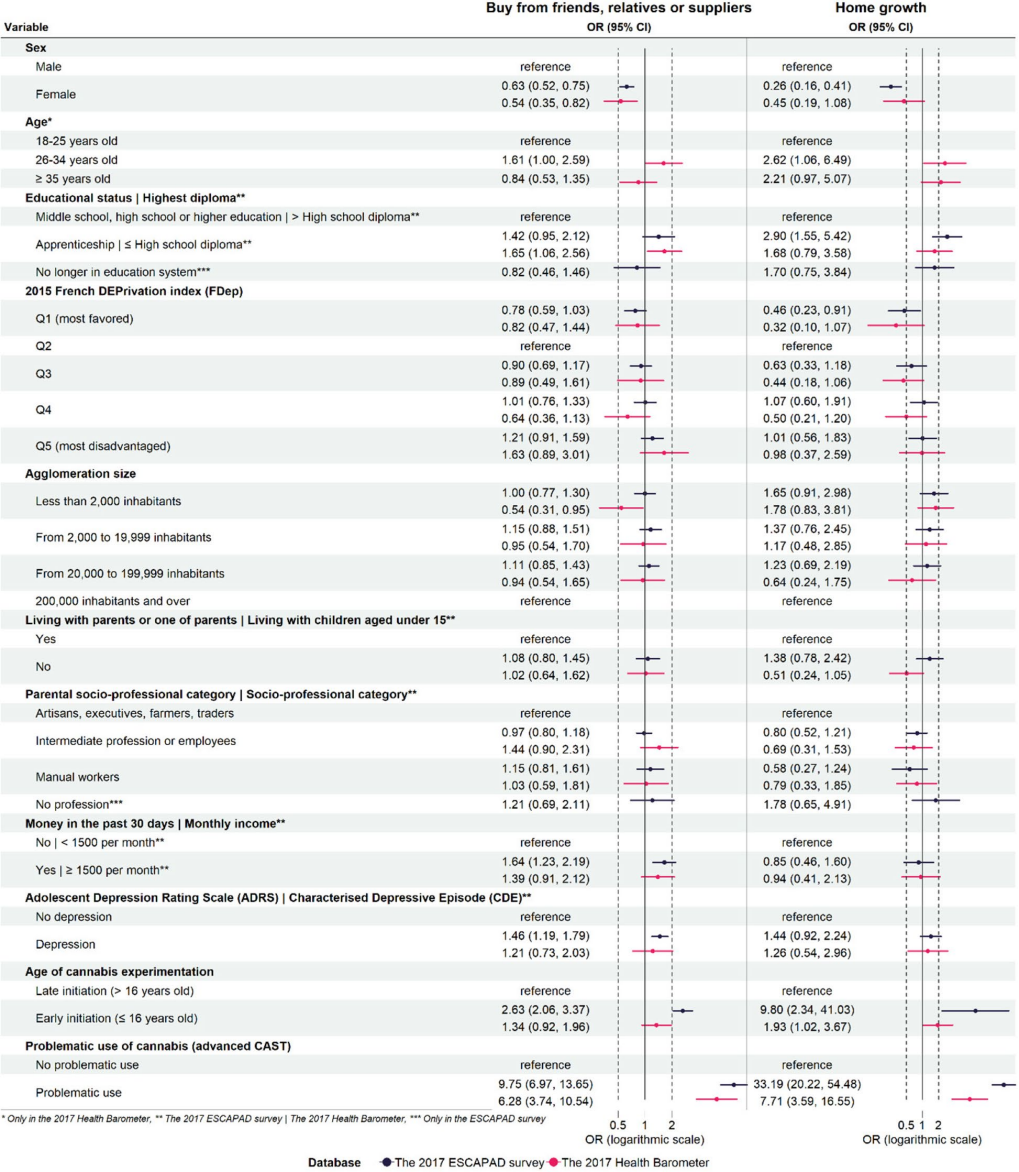
Multivariate multinomial regressions (ESCAPAD 2017, *n* = 2,943 | Health Barometer, *n* = 1,221) (reference: "Obtained for free")

Compared to obtaining cannabis for free, being male and experiencing problematic cannabis use were associated with both buying and home cultivation of cannabis. Among 17-year-olds, being in an apprenticeship was associated with a higher likelihood of home cultivation (OR = 2.90, 95%CI = 1.06–6.49), while earning money in the past 30 days and experiencing depression were associated with buying cannabis (OR = 1.64, 95%CI = 1.23–2.19, and OR = 1.46, 95%CI = 1.19–1.79, respectively). Early experimentation with cannabis was associated with both supply methods. Among adults, being aged 26–34 years was associated with home cultivation, while having less than a high school diploma was only associated with buying cannabis (Fig. 2).

### Sensitivity analyses

The first sensitivity analysis revealed minimal changes in the results, thereby reinforcing the methodology used to classify individuals according to the different methods of cannabis supply for the study of associated factors (Supplementary Table 1).

In the second sensitivity analysis, which distinguished between purchasing cannabis from friends or relatives and from suppliers in the 2017 ESCAPAD survey, both modes of supply exhibited similar factors associated with cannabis use. Combining these groups thus enhanced our statistical power while maintaining comparability with the Health Barometer data, without compromising the primary findings (Supplementary Table 2).

In the third sensitivity analysis, it was confirmed that by including people who had used cannabis throughout the year in ESCAPAD, we encompassed a significantly larger population, ensuring consistency without sacrificing statistical power (Supplementary Table 2).

## Discussion

Using two representative French national surveys, our study highlights differences among three cannabis supply methods (i.e., obtaining it for free, buying from friends, relatives or dealers, and home cultivation) and the various associated factors among both adolescents and adults. We demonstrated that women were less likely than men to buy or cultivate cannabis, compared to obtaining it for free. Conversely, individuals over 25 years old were more likely to cultivate cannabis than younger demographics. This trend also applies to apprentices, who were more likely to cultivate cannabis than those in middle school, high school, or higher education.

Our findings highlight sex disparities in cannabis supply methods, with males showing a greater propensity for buying or cultivating cannabis. This aligns with existing literature indicating that females are more likely to obtain cannabis from friends or family and less likely to acquire it from strangers or dealers (D’Amico et al. 2020; Vuolo & Matias 2020; Skliamis & Korf, 2022a). Additionally, males tend to spend more on cannabis, exhibit a higher frequency of use, and experience more symptoms and consequences of cannabis use disorders (D’Amico et al. 2020)*.* Similarly, previous studies have shown that men are more inclined to cultivate cannabis, whether in North American countries (Azofeifa et al. 2021; Cristiano et al. 2022) or European Countries (Vuolo & Mathias 2020; Bastien et al. 2025; Meisel et al. 2025). A French study reported an odds ratio (OR) of 2.61 (95%CI:1.86–3.66] for cannabis cultivation among men compared with women and other gender identities, with the main motivations being to control product quality and to avoid the illegal market (Bastien et al. 2025). Nevertheless, the gender gap appears to be narrowing, with more women entering cannabis cultivation, especially in legal contexts, and the odds of cultivating in illegal contexts were 1.49 times higher for men than for women (Meisel et al. 2025). In a qualitative study, interviews findings illuminate the concept of "gendered accessibility" within the cannabis market. It was observed that women generally access cannabis in social gatherings where it is shared by men, often through non-monetary social exchanges. Men are seen as suppliers, while women are positioned as recipients, potentially linked to a lower frequency of consumption (Kolar 2021)*.* However, this could also be attributed to men having greater access to substances compared to women (McHugh et al. 2018). Indeed, women are often portrayed as more vulnerable buyers who tend to avoid direct transactions with dealers when acquiring cannabis (Kolar 2021). Nevertheless, due to the significantly rise of delivery services in France since 2010, purchasing cannabis has become safer than buying it on the street (OFDT, ND). As a result, female users may have become more independent from men when it comes to procuring cannabis themselves.

The methods of cannabis supply are also influenced by age. We found that the majority of 17-years-old, despite a median age of cannabis initiation of 16.0 years (Hayatbakhsh et al. 2013), obtained their cannabis through purchases. A recent study indicated that adolescents who bought cannabis were more likely to engage in frequent cannabis use six months later (Kelleghan et al. 2022). Among adults, the association between being 26–34 years old and engaging in home cultivation aligns with previous research, which has shown that home growers are typically individuals between the ages of 20 and 40 (Cristiano et al. 2022).

Existing evidence shows that low educational attainment and disadvantaged socioeconomic status are associated with greater vulnerability to illicit drug use and a greater reliance on informal and riskier supply channels. Individuals in precarious social situations are more likely to rely on visible, street-based markets due to limited access to protected social networks and private settings for procurement (Room 2005; Seddon 2006; Babor et al. 2010). These structural constraints shape not only patterns of use but also modes of supply and exposure to law enforcement. Our findings are consistent with this literature. Among 17-year-olds, we observed a significant association between being an apprentice, a status often associated with lower educational attainment and more disadvantaged socioeconomic background, and engagement in home cannabis cultivation, compared with high school students. To our knowledge, this association has not been previously reported in international studies and therefore represents a novel contribution. This association may be partly explained by greater autonomy and access to resources among apprentices, which could influence their decision to cultivate cannabis, regardless of their urban environment. In addition, individuals with higher levels of education may perceive greater risks associated with cannabis cultivation because of better knowledge of legal frameworks and potential consequences. Conversely, apprentices may be less aware of these risks or perceive cannabis cultivation as less risky. Apprentices may also display higher levels of cannabis use, which could contribute to a greater likelihood in cannabis cultivation, given the observed association between cultivation and more intensive use. Our findings suggest that apprentices tend to report more frequent and problematic use than students in general or higher education. Cannabis cultivation may also reflect a strategy to access cannabis while minimizing exposure to street-based markets and potential arrest. Indeed, people with lower educational attainment and modest economic status are disproportionately exposed to processes of stigmatization and criminalization (Observatoire des inégalités, 2023). People from disadvantaged backgrounds are more frequently framed in public and media discourse as 'delinquents' or 'traffickers' rather than as users, reinforcing social stereotypes and legitimizing more repressive policing strategies. Arrests and convictions for drug use or possession can have long-lasting consequences, including criminal records, reduced access to employment and housing, and social marginalization, disproportionately affecting already vulnerable populations (Simckes et al., 2021; Bacon, 2022). Although then introduction of fixed fines for drug use may appear to soften repression, such measures may in practice reinforce social inequalities. Individuals with greater financial resources can more easily pay fines, whereas those with limited means are more vulnerable to escalating sanctions, debt, or judicial proceedings in the event of non-payment. Furthermore, disadvantaged users are more likely to purchase cannabis in public spaces or through visible informal networks, increasing their exposure to police surveillance and controls. Numerous studies have shown that police checks and arrests for cannabis-related offenses are disproportionately concentrated in working-class and racialized neighborhoods (Blanchard 2014; Vie publique 2025; Gunadi & Shi 2022; Shiner et al 2017). Taken together, these mechanisms may help explain why apprentices were almost three times more likely to report cannabis cultivation than individuals in general education.

The association between earning money in the past 30 days and cannabis purchases among 17-year-olds highlights that one of the main reasons for growing cannabis is cost savings (Alvarez et al. 2016; Potter et al. 2015). Furthermore, among adults, the association between having a high school diploma or less and buying cannabis underscores the role of educational attainment in influencing procurement choices. Conversely, Cristiano et al. demonstrated an association between cultivating cannabis and having more than a high school diploma (Cristiano et al. 2022)*.* However, their study examined cannabis cultivation both before and after legalization in Canada, with data collected just 10 months before the law changed. This suggests that anticipation of legalization may have influenced cultivation practices.

We also found an association among adolescents between symptoms of depression and purchasing cannabis. Some young individuals may use cannabis to cope with stress, anxiety, or depression, or to self-medicate psychiatric symptoms (Whiteley et al. 2021). A perceived need for cannabis may lead individuals to prioritize purchasing it, especially since obtaining cannabis for free is often context-dependent, such as during social gatherings. Buying cannabis may also be more practical than growing it, especially for young people who lack the resources, space, or knowledge required for cultivation. Additionally, young individuals experiencing depressive symptoms may opt to buy cannabis as a quicker option to obtain immediate relief from their symptoms. However, this coping strategy may prove counterproductive in the long term and exacerbate symptoms of depression (Gobbi et al. 2019; Lev-Ran et al. 2014). Furthermore, depression can lead individuals to engage in risky behaviors (Johnstad 2024), including the illegal purchase of cannabis. Young people with depressive symptoms may be more likely to associate with peers who already use cannabis, who may facilitate or encourage purchasing. Social group influence can play an important role in substance use choices (Torrejón-Guirado et al. 2023).

Finally, the association between early cannabis experimentation among adolescents and engagement in both buying and home cultivation suggests that initial experiences and the duration of cannabis use may play a crucial role in shaping individuals' future procurement preferences. The association between problematic cannabis use and a higher likelihood of buying or cultivating cannabis aligns with previous findings in the literature, notably that cannabis cultivation is predictive of increased cannabis use frequency (Azofeifa et al. 2021), and daily cannabis use (Cristiano et al. 2022). This finding also supports one of our hypotheses, which posits that the procurement of cannabis follows a trajectory: initially experimenting with friends, sharing, and obtaining it for free; then, in the case of continued consumption, purchasing one's own cannabis; and finally, cultivating it, after becoming familiar with it, particularly for personal use, cost-saving, avoiding contact with illegal sources, or controlling its quality (Alvarez et al. 2016; Potter et al. 2015). Nevertheless, the method of supply is not neutral; it exposes users to various types of harm. Purchasing from a street dealer carries a high risk of arrest, fines, or imprisonment. While still illegal, buying from friends or family is generally less risky. Home cultivation involves fewer day-to-day risks but may result in more severe legal consequences, depending on the quantity involved. However, self-cultivation offers greater control over product quality, unlike purchases from unknown sources, which may pose health risks due to uncertain composition. Despite its potential normalization, cannabis is still perceived as deviant, but social judgment varies according to gender, age, or social status (Borojevic & Söhner 2025; Dahlke et al. 2024)). For example, a woman or an elderly person buying cannabis on the street may face greater stigmatization and negative judgment (Hathaway et al. 2016; Skliamis et al., 2022b; Dahlke et al 2025). Therefore, the choice of supply method depends on multiple factors and is not solely determined by consumption frequency. Understanding these perceived and actual risks is essential for evaluating their impact on public health, harm reduction, and cannabis regulation.

### Limitations and strengths

Our study had several limitations. First, one of them is its cross-sectional nature, which inherently lacks the ability to track changes over time among respondents. Additionally, focusing solely on data from a single year (2017) precludes any analysis of trends. Moreover, the data are somewhat outdated, which slightly limits the scope of the results. Nevertheless, these are the most recent quantitative data available for France, and major changes in the cannabis market primarily occurred between 2000 and 2010, well before our study. Indeed, self-cultivation has become increasingly popular since the 2000 s, with a boom in the 2010 s, driven by greater availability of seeds, online tutorials, and technologies such as LED lights. Furthermore, although purchasing cannabis online dates back to the early days of e-commerce, it gained significant momentum with the development of the Dark Web and cryptocurrencies, particularly with the emergence of Silk Road in 2011 (Martin 2014). Following the closure of several major Dark Web marketplaces, alternatives platforms have proliferated, leading to a rise in smaller-scale platforms. Since 2016, social networks and messaging apps (Telegram, Instagram, Snapchat) have become increasingly popular channels for selling cannabis, often facilitating local deliveries (Berg et al. 2023; Nali et al. 2024). Although the data were collected in 2017, several elements support their continued relevance. The structural organization of the cannabis market in France appears to have remained relatively stable, with peer networks, street-level dealers, and informal sharing continuing to predominate (Salhi 2025). No major legal reform have fundamentally altered the market since that time. While home delivery services expanded during the COVID-19 pandemic, these developments appears to reflect adaptations of existing informal markets rather than structural transformation (Gérome 2023). Public perceptions have evolved, with people who use cannabis increasingly viewed as less dangerous (50% in 2018 vs. 60% in 2013) and public opinion divided regarding legalization (45% in favor in 2018, 66% among people who have ever used cannabis and 29% among those who have never used it) (Spilka et al. 2019). However, the broader policy context remains largely unchanged, with the governing majority and the Ministry of the Interior maintaining opposition to decriminalization and legalization (Poivret 2025). The Ministry of Interior has launched campaigns aimed at stigmatizing consumers (Rédaction & AFP, 2025) and has carried out numerous “clean sweep” crackdowns on drug-dealing areas in cities, with limited effectiveness (Albertini 2024; Mazerolle 2024). In this context, the 2017 findings remain a relevant reference for understanding current patterns of cannabis acquisition in France. Second, limiting our analysis to the departmental level prevents us from accessing more granular data, which could provide more nuanced insights, such as at the communal level. However, the French DEPrivation index (FDep) has been shown to be a reliable geographical indicator of socioeconomic conditions (Rey et al. 2009; Windenberger et al. 2012). Third, In the ESCAPAD dataset, the level of detail regarding different procurement methods was simplified to allow comparisons between the two databases. Nevertheless, we conducted a sensitivity analysis to further examine these procurement methods, and the results remained consistent. In the Health Barometer, questions regarding supply methods were only posed to consumers who have used cannabis in the past month, which reduced the sample size and restricted the analyses to current users. However, parallel analyses using ESCAPAD data produced similar results. Fourth, categorizing individuals into one of three procurement methods (receiving for free, purchasing, or cultivating) overlooks the nuances and complexity of social networks and acquisition patterns. Given the vast number of possible combinations, it was not feasible to analyze all of them due to statistical power constraints. Instead, we examined the broader consumption chain to identify different user profiles within it. In future studies with larger sample sizes, exploring all possible procurement combinations would be valuable. Fifth, the context and setting in which the ESCAPAD questionnaire was administered, namely during the mandatory National Defense and Citizenship Day (NDCD), in collaboration with the Army’s National Service Bureau, could have introduced bias and led to underreporting of psychoactive drug use. Nevertheless, to minimize this impact, questionnaires were completed anonymously and placed in envelopes that were sealed by the participants themselves and sent directly to the researchers. In addition, the study presentation emphasized that it was independent of the NDCD and guaranteed the anonymity and confidentiality of responses, in order to reassure respondents about the purpose and use of the data. Furthermore, announcing that some results from the previous survey would be shared after questionnaire completion helped present the survey as an “exchange” of information rather than a one-way data collection, thereby highlighting the value of the data and its future use. For the Health Barometer, as respondents were interviewed by investigators, there was a potential for social desirability bias, with possible underreporting of substance use. Nevertheless, interviewers’ training to use neutral, non-judgmental tone and to strictly follow standardized scripts may have limited its impact. In addition, the absence of face-to-face contact may have reduced perceived judgment and social pressure, and participants were explicitly informed about the confidentiality of their responses. Telephone-based surveys are widely used in epidemiological research on sensitive health behaviors, including substance use and are considered an acceptable method for collecting such data. Finally, as the study population consisted of individuals who used cannabis and reported using at least one cannabis supply method, any underreporting bias would have affected the statistical power of the results rather than the results themselves.

To highlight the strengths of our study, it is important to emphasize the representativeness of the French population within our sample. The study is based on data collected and weighted using rigorous methodologies, including standardized survey instruments, appropriate statistical techniques, and careful consideration of potential biases, ensuring the reliability and validity of the results. Additionally, by comparing data from multiple sources, the study provides a more comprehensive understanding of cannabis use behavior. It integrates data from 17-year-olds, an age corresponding to the typical initiation of cannabis use, with data from the entire French population aged 18–64 within the same year. By studying both age groups, we can identify specific risk factors associated with cannabis use in young people and older adults. This insight can contribute to the development of targeted prevention and intervention strategies for each age group, particularly for individuals at higher risk within specific cannabis supply methods. Tailored information sessions could be organized for each target group. To date, no effective interventions differentiate or personalize approaches based on how cannabis is obtained. However, interventions could, for example, target home growers by highlighting potential health risks and legal implications. Currently, only general drug prevention programs, such as the LifeSkills Training program, have proven successful. This program focuses on preventing early drug use among teenagers and promoting healthy alternatives to risky behaviors (Teyhan et al. 2016). The comparative approach used in this research adds depth to the analysis, strenghtens the validity of the conclusions, and provides a comprehensive overview of cannabis supply methods at different stages of life. Furthermore, our analyses account for a range of socio-economic and geographical factors, further enhancing the depth and reliability of our findings. Finally, it is important to note that these results were obtained in a country where cannabis remains illegal, as is the case in most countries worldwide.

## Conclusion

Knowledge of the factors associated with cannabis supply patterns can have several important implications for public health and harm reduction. It enables a better understanding of real-world practices, supports risk prevention, informs the adaptation of public policies, and contributes to the protection of populations most exposed to problematic use or high-risk supply channels, even in the absence of a legal framework. Identifying the profiles most likely to purchase cannabis through various channels makes it possible to tailor information campaigns and prevention messages, as well as to target interventions such as harm reduction programs, the distribution of cannabis testing kits, access to reliable information, and practical advice (e.g., avoiding street purchases, paying attention to product appearance, etc.). Even without legalization, harm reduction strategies can be implemented, for example, by educating users on the risks of contaminated products, promoting moderate consumption, and ensuring access to support services. These measures help reduce health risks associated with the uncertain quality of illicit cannabis, which tends to be lower in the case of home cultivation. Certain groups (e.g., young people, marginalized populations, regular users) appear to rely more frequently on riskier supply methods. Understanding this allows for the implementation of targeted actions, such as school-based interventions, psychosocial support, and mobile outreach or screening services. Similarly, understanding the socio-economic and contextual factors that influence sourcing methods (e.g., living in disadvantaged neighborhoods, peer pressure) can help to better tailor social, educational, and health policies. This may also contribute to preventing youth involvement in criminal networks. Moreover, understanding who purchases cannabis could help identify financial flows, distribution networks, and key players in the black market.

There can be many reasons for choosing one method of supply over another. These may include: 1- Economic factors, as self-cultivation is less expensive for heavy users; 2- The supplier’s reputation and the consumer’s trust in the seller, with many consumers obtaining cannabis from friends or acquaintances perceived to be safer; 3- The type of product sought, particularly if the consumer is looking for a specific potency; 4- The perceived risks associated with each mode of supply, as self-cultivation allows for quality control and helps avoid contact with criminal networks. Although illegal, it is considered by some to be less risky from a legal standpoint than direct purchase; 5- Individual motivation, particularly the desire for discretion: the more a person wants to stay "under the radar," the more they will seek out indirect channels; 6- Geographical factors, such as whether one lives in an urban or rural area, with access often being easier in cities; 7- Contextual factors, especially in areas where cannabis purchasing is associated with violent or criminal networks, which may deter some consumers; 8- user characteristics, for example, women may be more reluctant to purchase cannabis from a trap house due to safety concerns. Future research, particularly qualitative studies, should aim to explore why people who use cannabis make certain supply choices and how these decisions are shaped by a social and legal context marked by prohibition, criminalization, stigmatization, and social sanction.

This study fills a gap in the existing literature by examining cannabis supply methods among young people and adults in France. The data can guide policy reforms and support the development of more appropriate alternatives, such as decriminalization or controlled legalization. Certain practices, such as self-cultivation or sharing, may persist even after legalization. Understanding these dynamics allows for better anticipation and response to evolving trends. Nevertheless, future research would benefit from analyzing changes in supply methods over time while considering demographic and territorial factors.

## Supplementary Information

Supplementary Material 1.

## Data Availability

The datasets used in the current study are available upon reasonable request from the National Public Health Agency (Santé Publique France) for the Health Barometer survey and from the French Monitoring Centre on Drugs and Drug Addiction (Observatoire français des drogues et des tendances addictives—OFDT) for the ESCAPAD survey. All analyses are available from the corresponding author.
