# Supplementary material for: Factors associated with different cannabis supply methods: results from the French 2017 ESCAPAD and Health Barometer surveys
Source: J Cannabis Res. 2026 Jan 8;8:19. doi: 10.1186/s42238-025-00372-x (PMC12874936; doi:10.1186/s42238-025-00372-x)
Supplement: Supplementary file 1 — Supplementary Material 1. [file 42238_2025_372_MOESM1_ESM.docx]

**Supplementary Table 1.** Sensitivity analysis 1: Individuals who *bought cannabis from friends, relatives, or suppliers* according to the priority result *(Home cultivation > Bought from friends, relatives, or suppliers > Obtained for free)* but who had bought only once or twice and had also *obtained it for free*, were classified in this category

**Multivariate multinomial regressions (ESCAPAD n=2,943; Health Barometer n = 1,221; reference: "Obtained for free")**

|  | **The 2017 ESCAPAD survey** | | | | **The 2017 Health Barometer** | | | |
| --- | --- | --- | --- | --- | --- | --- | --- | --- |
|  | Bought from friends, relatives or suppliers (n = 1,540) | | Home cultivation (n = 151) | | Bought from friends, relatives or suppliers  (n=772) | | Home cultivation  (n=96) | |
|  | OR (95% CI) | p-value | OR (95% CI) | p-value | OR (95% CI) | p-value | OR (95% CI) | p-value |
| **Sex** |  |  |  |  |  |  |  |  |
| Male | reference |  | reference |  | reference |  | reference |  |
| Female | 0.59 (0.50, 0.71) | **<0.001** | 0.25 (0.16, 0.40) | **<0.001** | 0.60 (0.41, 0.90) | **0.013** | 0.51 (0.21, 1.20) | 0.121 |
| **Age*** |  |  |  |  |  |  |  |  |
| 18-25 years old |  |  |  |  | reference |  | reference |  |
| 26-34 years old |  |  |  |  | 1.44 (0.93, 2.24) | 0.101 | 2.35 (0.97, 5.71) | 0.058 |
| ≥ 35 years old |  |  |  |  | 0.96 (0.61, 1.53) | 0.874 | 2.44 (1.07, 5.54) | **0.033** |
| **Educational status \| Highest diploma**** |  |  |  |  |  |  |  |  |
| Middle school, high school or higher education \| > High school diploma** | reference |  | reference |  | reference |  | reference |  |
| Apprenticeship \| ≤ High school diploma** | 1.60 (1.08, 2.38) | **0.019** | 3.01 (1.61, 5.62) | **0.001** | 1.59 (1.06, 2.40) | **0.026** | 1.61 (0.77, 3.37) | 0.204 |
| No longer in education system*** | 0.80 (0.45, 1.43) | 0.452 | 1.73 (0.78, 3.80) | 0.175 |  |  |  |  |
| **2015 French DEPrivation index (FDep)** |  |  |  |  |  |  |  |  |
| Q1 (most favored) | 0.81 (0.62, 1.06) | 0.130 | 0.49 (0.25, 0.96) | **0.037** | 0.74 (0.44, 1.25) | 0.256 | 0.30 (0.09, 0.98) | **0.046** |
| Q2 | reference |  | reference |  | reference |  | reference |  |
| Q3 | 0.87 (0.67, 1.14) | 0.312 | 0.62 (0.33, 1.17) | 0.143 | 0.79 (0.45, 1.39) | 0.419 | 0.41 (0.17, 0.97) | **0.042** |
| Q4 | 1.02 (0.78, 1.35) | 0.870 | 1.08 (0.61, 1.92) | 0.787 | 0.64 (0.36, 1.13) | 0.125 | 0.51 (0.21, 1.23) | 0.133 |
| Q5 (most disadvantaged) | 1.21 (0.92, 1.58) | 0.174 | 0.99 (0.55, 1.77) | 0.962 | 1.38 (0.78, 2.47) | 0.272 | 0.86 (0.33, 2.21) | 0.749 |
| **Agglomeration size** |  |  |  |  |  |  |  |  |
| Less than 2,000 inhabitants | 0.98 (0.76, 1.27) | 0.898 | 1.62 (0.90, 2.92) | 0.108 | 0.56 (0.32, 0.97) | 0.038 | 1.84 (0.87, 3.89) | 0.108 |
| From 2,000 to 20,000 inhabitants | 1.32 (1.02, 1.71) | **0.038** | 1.50 (0.84, 2.67) | 0.173 | 0.88 (0.50, 1.53) | 0.638 | 1.06 (0.44, 2.57) | 0.892 |
| From 20,000 to 200,000 inhabitants | 1.04 (0.80, 1.34) | 0.782 | 1.18 (0.66, 2.10) | 0.580 | 1.02 (0.60, 1.73) | 0.952 | 0.68 (0.25, 1.82) | 0.440 |
| More than 200,000 inhabitants | reference |  | reference |  | reference |  | reference |  |
| **Living with parents or one of parents \| Living with children aged under 15**** |  |  |  |  |  |  |  |  |
| Yes | reference |  | reference |  | reference |  | reference |  |
| No | 0.95 (0.72, 1.26) | 0.715 | 1.25 (0.72, 2.17) | 0.435 | 0.94 (0.61, 1.46) | 0.786 | 0.48 (0.23, 0.97) | **0.042** |
| **Parental socio-professional category \| Socio-professional category**** |  |  |  |  |  |  |  |  |
| Artisans, executives, farmers, traders | reference |  | reference |  | reference |  | reference |  |
| Intermediate professions or employees | 0.97 (0.80, 1.17) | 0.725 | 0.84 (0.55, 1.27) | 0.410 | 1.45 (0.92, 2.27) | 0.108 | 0.69 (0.31, 1.50) | 0.343 |
| Manual workers | 1.27 (0.91, 1.77) | 0.157 | 0.73 (0.35, 1.53) | 0.406 | 1.17 (0.68, 2.00) | 0.568 | 0.86 (0.38, 1.98) | 0.732 |
| No profession*** | 1.45 (0.81, 2.59) | 0.215 | 2.22 (0.80, 6.13) | 0.125 |  |  |  |  |
| **Money in the past 30 days \| Monthly income**** |  |  |  |  |  |  |  |  |
| No \| < 1500 per month** | reference |  | reference |  | reference |  | reference |  |
| Yes \| ≥ 1500 per month** | 1.45 (1.08, 1.95) | **0.013** | 0.76 (0.40, 1.41) | 0.378 | 1.37 (0.92, 2.05) | 0.121 | 0.92 (0.41, 2.06) | 0.835 |
| **Adolescent Depression Rating Scale (ADRS) \| Characterised Depressive Episode (CDE)**** |  |  |  |  |  |  |  |  |
| No depression | reference |  | reference |  | reference |  | reference |  |
| Depression | 1.35 (1.10, 1.65) | **0.004** | 1.36 (0.88, 2.10) | 0.169 | 1.20 (0.75, 1.94) | 0.447 | 1.24 (0.54, 2.85) | 0.611 |
| **Age of cannabis experimentation** |  |  |  |  |  |  |  |  |
| Late initiation (> 16 years old) | reference |  | reference |  | reference |  | reference |  |
| Early initiation (≤16 years old) | 2.35 (1.82, 3.03) | **<0.001** | 8.60 (2.07, 35.71) | **0.003** | 1.41 (0.98, 2.02) | 0.064 | 1.97 (1.05, 3.70) | **0.034** |
| **Problematic use of cannabis (advanced CAST)** |  |  |  |  |  |  |  |  |
| No problematic use | reference |  | reference |  | reference |  | reference |  |
| Problematic use | 10.59 (7.87, 14.25) | **<0.001** | 31.30 (19.61, 49.98) | **<0.001** | 6.51 (4.08, 10.4) | **<0.001** | 7.39 (3.54, 15.4) | **<0.001** |

* Only in the 2017 Health Barometer, ** The 2017 ESCAPAD survey | The 2017 Health Barometer, *** Only in the ESCAPAD survey

**Supplementary Table 2.** Sensitivity analysis 2 & 3 for ESCAPAD - Multivariate multinomial regressions

|  | Sensitivity analysis 2: **Separate bought from friends and dealers** *reference: "Obtained for free" (n=1,014)* | | | | | | Sensitivity analysis 3: **Select monthly users** *reference: "Obtained for free" (n=525)* | | | |
| --- | --- | --- | --- | --- | --- | --- | --- | --- | --- | --- |
|  | Bought from friends, or relatives (n = 571) | | Bought from suppliers (n = 1,207) | | Home cultivation (n = 151) | | Bought from friends, relatives or suppliers (n = 1,457) | | Home cultivation (n = 136) | |
|  | OR (95% CI) | p-value | OR (95% CI) | p-value | OR (95% CI) | p-value | OR (95% CI) | p-value | OR (95% CI) | p-value |
| **Sex** |  |  |  |  |  |  |  |  |  |  |
| Male | reference |  | reference |  | reference |  | reference |  | reference |  |
| Female | 0.80 (0.64, 1.00) | **<0.001** | 0.52 (0.42, 0.64) | **<0.001** | 0.23 (0.15, 0.37) | **<0.001** | 0.59 (0.47, 0.75) | **<0.001** | 0.25 (0.15, 0.42) | **0.048** |
| **Educational status** |  |  |  |  |  |  |  |  |  |  |
| Middle school, high school or higher education | reference |  | reference |  | reference |  | reference |  | reference |  |
| Apprenticeship | 1.50 (0.93, 2.43) | 0.545 | 1.32 (0.85, 2.06) | 0.218 | 2.63 (1.37, 5.05) | **0.004** | 1.17 (0.71, 1.91) | **0.011** | 2.52 (1.24, 5.12) | 0.094 |
| No longer in education system*** | 0.98 (0.50, 1.94) | 0.230 | 0.73 (0.38, 1.41) | 0.349 | 1.64 (0.72, 3.76) | 0.239 | 0.63 (0.30, 1.34) | 0.439 | 1.45 (0.56, 3.74) | 0.958 |
| **2015 French DEPrivation index FDep)** |  |  |  |  |  |  |  |  |  |  |
| Q1 (most favored) | 0.78 (0.55, 1.10) | 0.123 | 0.80 (0.59, 1.09) | 0.152 | 0.47 (0.24, 0.94) | **0.032** | 0.76 (0.54, 1.08) | **0.035** | 0.45 (0.21, 0.94) | 0.158 |
| Q2 | reference |  | reference |  | reference |  | reference |  | reference |  |
| Q3 | 0.98 (0.71, 1.37) | 0.404 | 0.85 (0.62, 1.15) | 0.282 | 0.62 (0.33, 1.17) | 0.142 | 1.16 (0.82, 1.65) | 0.679 | 0.87 (0.44, 1.72) | 0.925 |
| Q4 | 0.98 (0.70, 1.39) | 0.647 | 1.05 (0.77, 1.44) | 0.762 | 1.09 (0.61, 1.96) | 0.768 | 1.09 (0.76, 1.56) | 0.624 | 1.17 (0.62, 2.22) | 0.927 |
| Q5 (most disadvantaged) | 1.05 (0.74, 1.49) | 0.200 | 1.33 (0.98, 1.82) | 0.069 | 1.05 (0.57, 1.90) | 0.885 | 1.26 (0.88, 1.81) | 0.922 | 1.03 (0.53, 2.01) | 0.773 |
| **Agglomeration size** |  |  |  |  |  |  |  |  |  |  |
| Less than 2,000 inhabitants | 1.19 (0.86, 1.64) | 0.196 | 0.85 (0.63, 1.13) | 0.267 | 1.51 (0.83, 2.75) | 0.177 | 0.80 (0.57, 1.12) | 0.273 | 1.43 (0.75, 2.71) | 0.303 |
| From 2,000 to 20,000 inhabitants | 1.09 (0.78, 1.54) | 0.536 | 1.19 (0.88, 1.60) | 0.259 | 1.37 (0.76, 2.47) | 0.300 | 1.12 (0.79, 1.58) | 0.282 | 1.42 (0.75, 2.71) | 0.601 |
| From 20,000 to 200,000 inhabitants | 1.24 (0.90, 1.71) | 0.973 | 0.97 (0.73, 1.30) | 0.839 | 1.15 (0.64, 2.07) | 0.632 | 1.01 (0.72, 1.41) | 0.889 | 1.05 (0.55, 2.01) | 0.190 |
| More than 200,000 inhabitants | reference |  | reference |  | reference |  | reference |  | reference |  |
| **Living with parents or one parent** |  |  |  |  |  |  |  |  |  |  |
| Yes | reference |  | reference |  | reference |  | reference |  | reference |  |
| No | 0.92 (0.63, 1.34) | 0.672 | 1.14 (0.83, 1.58) | 0.413 | 1.41 (0.80, 2.49) | 0.231 | 1.09 (0.73, 1.61) | 0.205 | 1.51 (0.80, 2.85) | 0.663 |
| **Parental socio-professional category** |  |  |  |  |  |  |  |  |  |  |
| Artisans, executives, farmers, traders | reference |  | reference |  | reference |  | reference |  | reference |  |
| Intermediate professions or employees | 0.89 (0.70, 1.12) | 0.751 | 0.97 (0.78, 1.20) | 0.752 | 0.83 (0.54, 1.27) | 0.384 | 0.96 (0.75, 1.23) | 0.461 | 0.84 (0.53, 1.34) | 0.316 |
| Manual workers | 1.22 (0.80, 1.84) | 0.423 | 1.18 (0.81, 1.73) | 0.388 | 0.70 (0.33, 1.49) | 0.353 | 1.21 (0.76, 1.94) | 0.252 | 0.60 (0.25, 1.43) | 0.356 |
| No profession | 1.29 (0.61, 2.73) | 0.297 | 1.73 (0.88, 3.40) | 0.109 | 2.54 (0.88, 7.29) | 0.084 | 1.50 (0.70, 3.19) | 0.146 | 2.35 (0.74, 7.40) | 0.512 |
| **Money in the past 30 days** |  |  |  |  |  |  |  |  |  |  |
| No | reference |  | reference |  | reference |  | reference |  | reference |  |
| Yes | 1.43 (1.00, 2.05) | **0.006** | 1.78 (1.27, 2.51) | **0.001** | 0.88 (0.46, 1.65) | 0.683 | 1.67 (1.16, 2.42) | 0.565 | 0.82 (0.41, 1.62) | 0.050 |
| **Adolescent Depression Rating Scale (ADRS)** |  |  |  |  |  |  |  |  |  |  |
| No depression | reference |  | reference |  | reference |  | reference |  | reference |  |
| Depression | 1.26 (0.98, 1.63) | **0.005** | 1.52 (1.21, 1.91) | **<0.001** | 1.49 (0.96, 2.33) | 0.077 | 1.47 (1.12, 1.92) | 0.070 | 1.58 (0.96, 2.59) | 0.073 |
| **Age of cannabis experimentation** |  |  |  |  |  |  |  |  |  |  |
| Late initiation (> 16 years old) | reference |  | reference |  | reference |  | reference |  | reference |  |
| Early initiation (≤16 years old) | 2.25 (1.64, 3.08) | **<0.001** | 3.14 (2.31, 4.27) | **<0.001** | 10.27 (2.46, 42.90) | **0.001** | 3.18 (2.30, 4.39) | **0.004** | 18.34 (2.47, 136.32) | **<0.001** |
| **Problematic use of cannabis (advanced CAST)** |  |  |  |  |  |  |  |  |  |  |
| No problematic use | reference |  | reference |  | reference |  | reference |  | reference |  |
| Problematic use | 2.30 (1.52, 3.49) | **<0.001** | 15.02 (10.63, 21.20) | **<0.001** | 33.84 (20.58, 55.65) | **<0.001** | 8.54 (5.71, 12.77) | **<0.001** | 25.40 (14.53, 44.40) | **<0.001** |
